# Supplementary material for: Parallelized calculation of permutation tests
Source: Bioinformatics. 2020 Dec 8;36(22-23):5392–7. doi: 10.1093/bioinformatics/btaa1007 (PMC8016463; doi:10.1093/bioinformatics/btaa1007)
Supplement: btaa1007_Supplementary_Data [file btaa1007_supplementary_data.zip › supplement.pdf]

Supplement to "Parallelized calculation of permutation tests"

Markus Ekvall[1], Michael Höhle[2], and Lukas Käll[1]

[1]Science for Life Laboratory, School of Engineering Sciences in Chemistry, Biotechnology and Health, KTH – Royal Institute of Technology, Box 1031, 171 21 Solna, Sweden
[2]Department of Mathematics, Stockholm University, 106 91 Stockholm, Sweden

November 9, 2020

Algorithm S1 # cardinality-j subsets s.t. their elements sum is equal to s.

m: Scalar equal to the number of samples in x^A.
n: Scalar equal to the number of samples x^B.
$s_{\max}$: Scalar equal to the sum of the m largest values in x.
x: Concatenated vector of x^A and x^B with length $m+n$.
procedure NumberOfSubsets(m, n, $s_{\max}$, x)
    $x = [0] + x$ ▷ Include the empty set $\emptyset$ to x
    $m = m + 1$ ▷ Correct the indices for this inclusion.
    Allocate array $N_{old}[0...s_{\max}, 0...m]$ with zeros
    Allocate array $N_{new}[0...s_{\max}, 0...m]$ with zeros
    for $i = 1$ to $(m+n)+1$ **do**
        for $j = 1$ to $m+1$ **do**
            for $s = 0$ to $s_{\max}+1$ **do**
                if $s = (j-1) = 0$ **then** ▷ Sub-recursion 7
                    $N_{new}[s, j-1] = 1$
                else if $i < j$ **then** ▷ Sub-recursion 8
                    $N_{new}[s, j-1] = 0$
                else if $j > 1$ and $x[i-1] \leq s$ **then** ▷ Sub-recursion 5
                    $N_{new}[s, j-1] = N_{old}[s - x[i-1], j-2] + N_{old}[s, j-1]$
                else if $j > 1$ and $x[i-1] > s$ **then**
                    $N_{new}[s, j-1] = N_{old}[s, j-1]$
                end if
            end for
        end for
        $N_{old} = N_{new}$; ▷ Update N_{old} for next iteration.
    end for
    return $N_{new}[0...s_{\max}, m]$ ▷ By remark **??**: $N_{m+n}[s, m] = N(s, m)$
end procedure

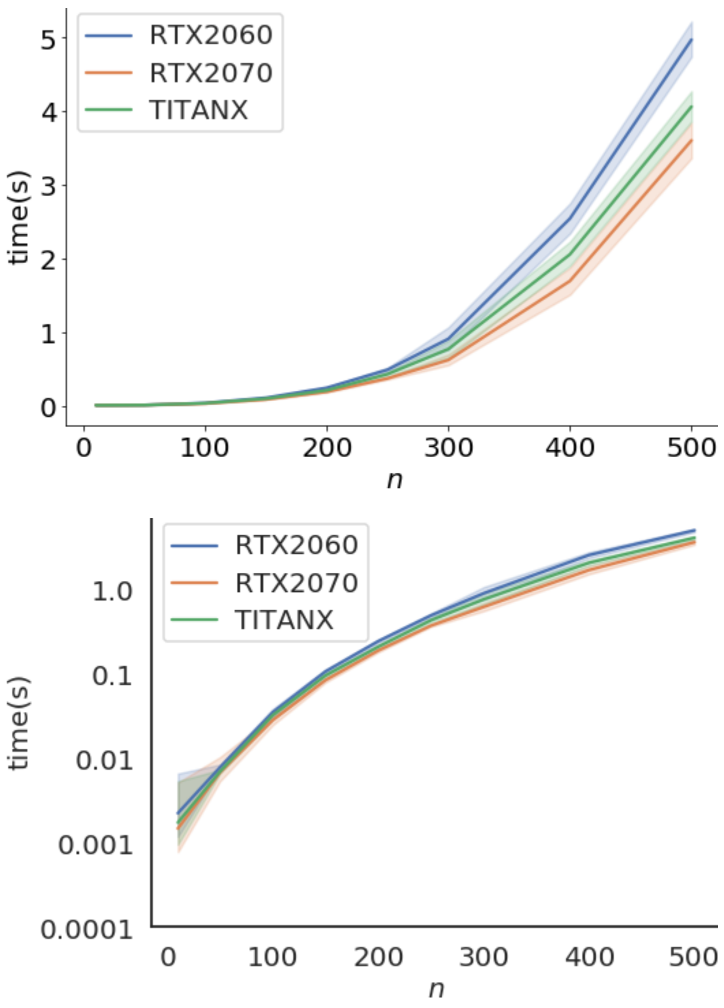

(a)

(b)

Figure S1: **Run time requirements of different GPUs**. We plotted the time required to calculate p values for samples from $y^A \sim \mathcal{N}(0.2, 1)$ and $y^B \sim \mathcal{N}(0.2, 1)$ for different sample sizes $\mathbf{n}$ on similar computers with different graphics cards. The mean time and the 95% confidence interval around the mean time to calculate each of 5 replicate samplings was plotted in (a) linear and (b) log scale. It should be noted that the execution times where highly reproducible, and it might be hard to see the confidence interval in the plots.

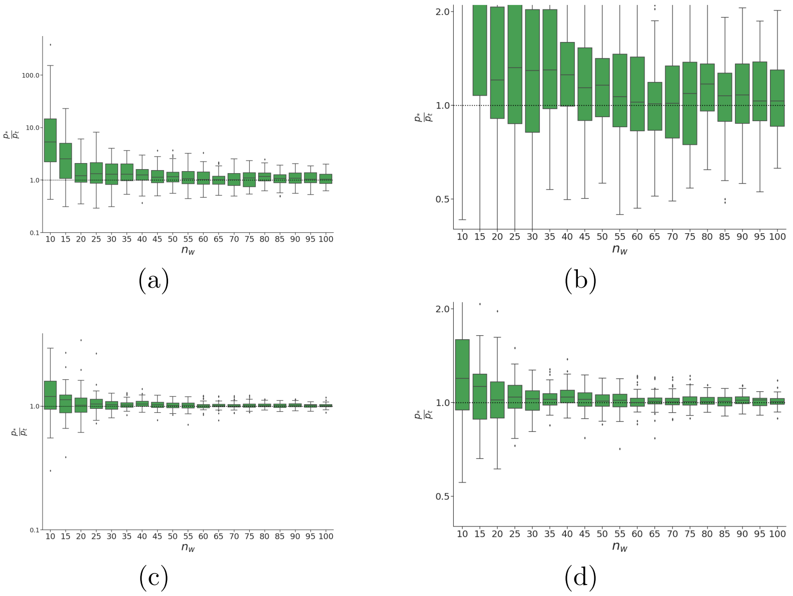

Figure S2: **The influence of n_w on the accuracy and precision of the Green algorithm.** We plotted the fold change difference between the p values calculated by the Green algorithm and a t test, as a function of the window size, n_w. Here we sampled 50 samples with sizes $|y^A| = |y^B| = 150$ from each of $y^A \sim \mathcal{N}(0.0, 1)$ and (a)(b) $y^B \sim \mathcal{N}(1.0, 1)$, or (c)(d) $y^B \sim \mathcal{N}(0.2, 1)$. Here we selected window sizes were $n_w \in \mathbf{n_w} = [10, 15, \dots, 95, 100]$. (a)(c) We plotted the full range and (b)(d) details of $\frac{p_*}{p_t}$ in the range $[0.4 - 2.1]$.

(a)

(b)

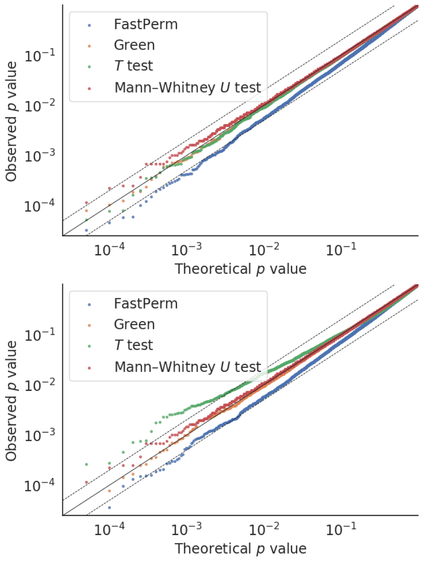

Figure S3: **Calibration plot, i.e. test of p values uniformity under null.** We compared the parallelized shift method, the Mann-Whitney U-test, and a t-tests uniformity under null. We tested 20000 samples with (a) $y_i^A, y_i^B \overset{\text{iid}}{\sim} \mathcal{N}(0,1)$ and (b) $y_i^A, y_i^B \overset{\text{iid}}{\sim} \log\mathcal{N}(0,1)$. In both cases the sample sizes were set to $|y_i^A| = |y_i^B| = 20$ and the number of discretization windows, n_w, were set to, 100. The continuous line $y = x$, and the dashed lines $y = 2x$ and $y = x/2$ were included as reference.
